# Association Between Future Orientation and Anxiety in University Students During COVID-19 Outbreak: The Chain Mediating Role of Optimization in Primary-Secondary Control and Resilience

**DOI:** 10.3389/fpsyt.2021.699388

**Published:** 2021-08-04

**Authors:** Yilin Chen, Hui Xu, Chuanshi Liu, Jing Zhang, Chenguang Guo

**Affiliations:** ^1^Department of Medical Imaging, The First Affiliated Hospital of Xi'an Jiaotong University, Xi'an, China; ^2^School of Psychology, Nanjing Normal University, Nanjing, China; ^3^Oriental Academy for Analytical psychology, City University of Macau, Macao, SAR China

**Keywords:** COVID-19, optimization in primary and secondary control, anxiety, future orientation, resilience

## Abstract

The unfamiliar and menacing epidemic has undoubtedly increased the anxiety of students. Therefore, the strategies to reduce anxiety are urgently required. The present study principally investigated a protective mechanism of future orientation in anxiety during the low-risk period of COVID-19 outbreak. The study was conducted in 528 non-infected students (range = 16–24 years) recruited from five universities in China. The participants completed questionnaires between January 22, 2021, and January 24, 2021. Chain intermediary analyses were performed after controlling for gender and age. Results indicated that future orientation lowers anxiety through (a) optimization in primary and secondary control, (b) resilience, and (c) chain mediating path of optimization in primary and secondary control coupled with resilience. We investigated how individuals deal with risk factors after encountering adversity and how their psychological flexibility stimulates and promotes them to achieve a well-adapted developmental state. This study provided reference suggestions on reducing anxiety levels during an emergency.

## Introduction

According to the expectancy model of fear (1), the events that shatter people's expectations trigger fear and anxiety, which has also been proven by studies on COVID-19 outbreak (2, 3). The self-efficacy theory (4) suggests that the people who are confident can cope with future events and are rarely anxious, which has been proven by a study on positive future orientations (5). However, in-depth studies are required to understand why future-oriented people exhibit lesser anxiety than other people and the underlying mechanism. Therefore, the aim of the present study was to investigate the relationship between future orientation and anxiety in non-infected students during COVID-19 outbreak. Future orientation for teenagers concretely includes planning (exploration and investment in future) and evaluation (emotional experience related to future education, career, and marriage goals). Considering that students' future orientation goals and concerns are most often associated with development tasks (6), educational and professional fields were targeted in this study.

People who aim for future and possess strong motivation to execute their plans are less anxious about existing emergencies (7). However, the motivation to realize future is weakened under challenging situations. Compared with the motivation, which is a temporary and highly fluctuating cognitive phenomenon, optimization in primary and secondary control scale (OPS) is a stable behavioral tendency derived from intrinsic motivation (8). The lifelong development view (9) suggests that the OPS, as a model of adjusting oneself with the external environment, optimizes, and maintains motivation by increasing either resource inputs or reliance on compensation strategies (10, 11), thereby reducing future anxiety (12–14). Therefore, we assumed that future orientation reduces anxiety by the mediation of OPS.

Although the effects of severe challenges that lead to stressful situations and setbacks of individual emotions have been proven, individuals do not experience negative emotions in a stressful situation alone. Positive emotions can be developed by constructing personal resources such as resilience (15–19). Resilience is not only a dynamic process between dangerous and protective characteristics (20) but also an individual's superior adaptability (21, 22). Moreover, it a result of the change process (23, 24). Organisms possess the essential response-ability of dynamic regulation and instant adaptation for self-protection and survival when the environment changes, which is a “self-regulation mechanism” determined by biological genetics (25). Resilience seems to be a self-protection instinct in humans (26) that help people in dealing with negative emotions (27, 28). Therefore, we assumed that future orientation could reduce anxiety by increasing resilience.

Notably, OPS and psychological resilience are not separate intermediaries. The process model of mental resilience (26) suggests that in response to life stimuli, protective factors either mobilize, reintegrate, and ultimately restore to maintain a balance or lose balance (15–19). Among these factors, OPS's support is a protective factor (29, 30). In the present study, we hypothesized that the future orientation could reduce anxiety through the chain mediation path of OPS and resilience.

**Figure 1:**
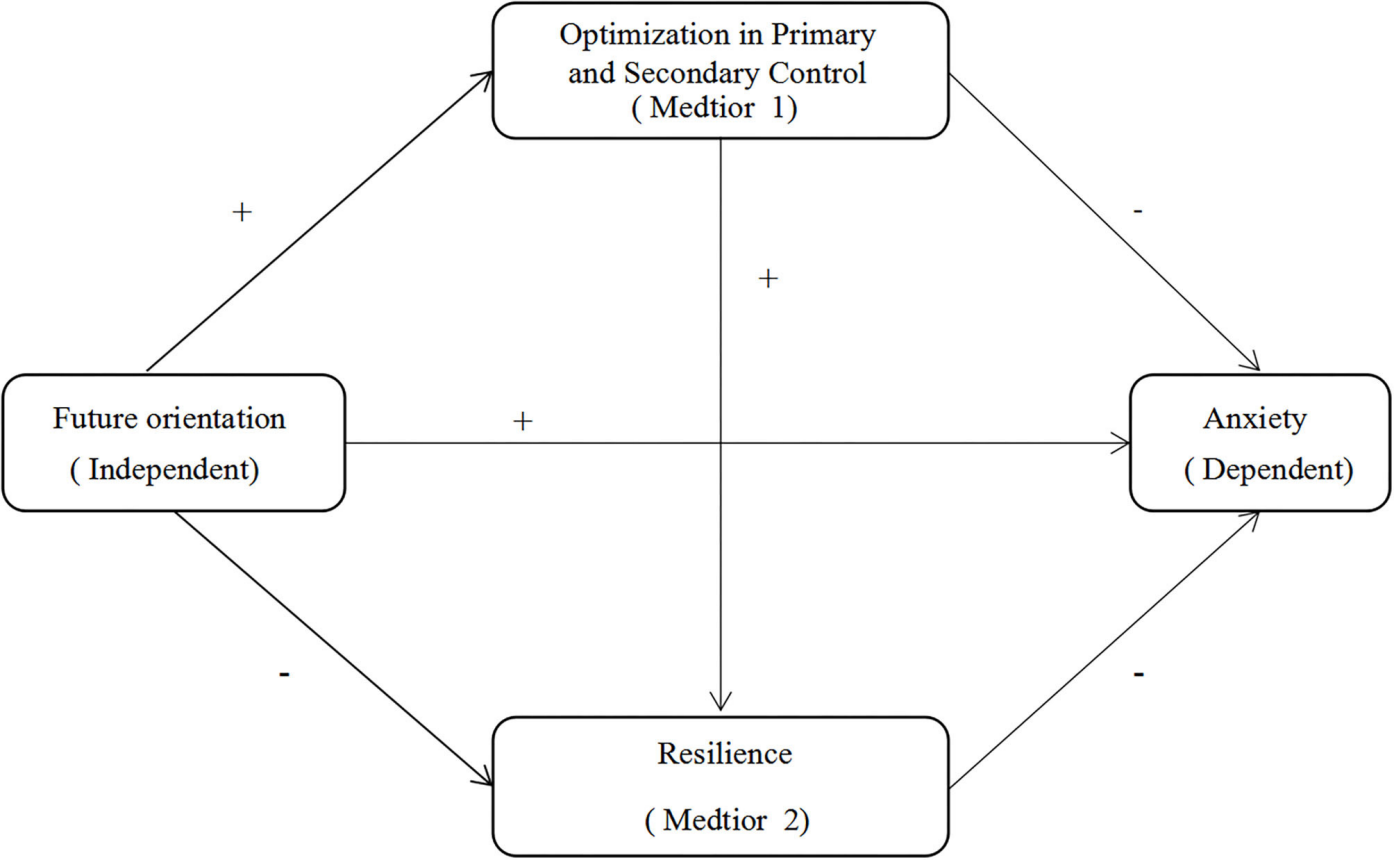
The proposed chain mediation model. Indirect effect 1, Future orientation → Optimization in primary and secondary control → Anxiety; Indirect effect 2, Future orientation → Resilience → Anxiety; Indirect effect 3, Future orientation → Optimization in primary and secondary control → Resilience → Anxiety.

Consequently, the study investigated the relationship between future orientation and anxiety in non-infected students during the low-risk period of COVID-19 outbreak. We propose three hypotheses in this study (Figure 1):

Future orientation could reduce anxiety through

H1: the mediating effect of OPS;
H2: the mediating effect of resilience;
H3: the chain mediating path between OPS and resilience.

## Materials and Methods

### Participants

Based on previous studies, over 500 participants were enrolled in this study (31–33). In this study, we have contacted each college secretary, obtained the oral consent of the secretary and class counselor, and informed all participants of the purpose of the study. We obtained the paper version of all the participants' informed consent, who were studying in five universities located in different cities of China. Study questionnaires were filled in by the recruited students between January 22, 2021, and January 24, 2021. During the mental health education course (~45 min), the research assistant told all students to fill out the paper questionnaire voluntarily with payment. After completion of the survey, the participants received 0.77 US dollars−2.3 US dollars as a bonus according to the consistency and completeness of their answers. The assistants also imported data entries to ensure that the data remains anonymous to the researchers.

Participants were required to fill in their sex (1 = male, 2 = female), their family's living status (1 = urban, 2 = rural), and their age and family structure. The age of all participants ranged from 16 to 24 years old (*M* = 19.16 years, SD = 0.94). The majority of all participants were from cities (about 77.98%, *n* = 412), with the remainder (about 22.02%, *n* = 116) were from towns. Among them, 93.75% of the subjects were from two-parent families (*n* = 495), included 266 males and 262 females. The author's research ethics committee approved the study.

### Connor–Davidson Resilience Scale

The Connor–Davidson Resilience Scale (CD-RISC), initially developed by Connor and Davidson, is a 25-item scale used to evaluate resilience (34), and it was modified into a Chinese version by Yu and Zhang (35). CD-RISC contains three subscales, namely strength (for example, can you cope with increasing pressure?), tenacity (for example, even if there is no hope, can you not give up easily?), and optimism (for example, can you see the other side of the coin?). CD-RISC is a 5-point Likert scale ranging from 0 (not correct at all) to 4 (almost always correct) that assesses resilience of participants. The final score is obtained by adding all items, and it ranges from 0 to 100; the higher the final score, the higher is the resilience. The Chinese version of CD-RISC exhibits superior reliability and validity (36), and was widely used in participants aged 13–30 (35, 37–41). It exhibited a stable internal consistency in the present study (Cronbach's alpha coefficient = 0.939).

### Future Orientation Questionnaire

Future orientation questionnaire (FQD) is mainly used to examine the development of future orientation of youths, and it was initially developed by Nurmi et al. (42). FQD with a 44-item scale mainly investigates the extent of future exploration (for example, have you often searched for information related to future education?) and investment (for example, did you prepare for your future objective?) in terms of aspects such as family, occupation, and education. This study investigated both the future educational (for example, do you often think about or plan your education you will receive in the future?) and occupational areas (for example, how determined are you to execute your future career plan after graduation?) of the participants. FQD uses a 5-point Likert scale ranging from 1 (never) to 5 (always) to assess the resilience of participants. The total score is the sum of the average scores of all items, with high total scores denoting a high future orientation level. The Chinese version of FQD shows good reliability and validity (6), and was widely used in participants aged 13–30 (6, 43). It demonstrated a stable internal consistency (Cronbach's alpha coefficient = 0.868) in the present investigation.

### Self-Rating Anxiety Scale

Self-rating anxiety scale (SAS), first developed by (44), is used to examine participants' anxiety symptoms over the preceding 7 days. It comprises 20 items and is scored by a 4-point Likert scale, ranging from 1 (no time or very little time) to 4 (absolutely most or all-time). A total SAS score of >50 indicates that the participant is more likely to develop anxiety symptoms. The Chinese version shows satisfactory reliability and validity (45). The self-rating anxiety scale is widely used in participants aged 15–24 (46, 47). In this study, the internal consistency of this questionnaire was stable (Cronbach's alpha coefficient = 0.881).

### OPS Scale

The OPS scale includes five subscales: selective primary control (for example, after setting a goal, I am willing to work hard to develop the skills required to achieve the goal), selective secondary control (for example, I can avoid any interference when I decide to do something), compensatory primary control (for example, when I cannot directly achieve a goal, I occasionally use a roundabout way to achieve it), compensatory secondary control (for example, when I am in difficult situations, I often comfort myself by telling that in many ways I am in a better position than others), and optimization (for example, if something takes a lot of time, I will consider whether I should invest). The 44-item OPS scale was designed by Heckhausen et al. (49) and further revised by Wang et al. (48). The first four subscales consist of eight items, and the optimization subscale comprises 12 questions. The total score is the sum of the average scores of all the items and is scored on the basis of a five-point scale (1–5). The higher the total score, the higher is the degree of optimization in primary and secondary controls (48, 49). The OPS scale is widely used in university students (50, 51). The Chinese version of OPS exhibits superior reliability and validity (48), and it demonstrated a strong internal consistency in the present study (Cronbach's alpha coefficient = 0.956).

### Statistical Analyses

SPSS 25.0 software was used for statistical analysis. First, perform descriptive statistics and Pearson correlation analysis on the research variables. Previous studies have found that some demographic factors, such as gender and age, are related to anxiety (52–54). Based on the above findings, we selected these variables as possible covariates in the subsequent analysis. Sex is a dichotomy variable (0 = male; 1 = female). Age is measured by the age of the respondent (in years).Statistical analyses were conducted using Model 6 of the PROCESS macro provided by (55), with future orientation as the independent variable, anxiety as the dependent variable, OPS and resilience as the intermediate chain variables, and controlling gender and age as covariance for examining the chain mediating effect of future orientation and anxiety. Moreover, 5,000 bootstrap samples with 95% confidence intervals were conducted to calculate the significance of indirect effects.

## Results

### Correlations Among All Variables

Table 1 presents the outcomes of the Pearson correlation test. A negative correlation was observed between anxiety and future orientation (*r* = −0.17, *p* < 0.01). OPS and future orientation were found to have a positive correlation (*r* = 0.34, *p* < 0.01); however, OPS was found to be negatively correlated with anxiety (*r* = −0.29, *p* < 0.01). Resilience was found to be positively correlated with future orientation (*r* = 0.46, *p* < 0.01) and negatively correlated with anxiety (*r* = −0.38, *p* < 0.01). Moreover, OPS displayed a positive correlation with resilience (*r* = 0.49, *p* < 0.01).

**Table 1:** Correlations among different variables (*N* = 528).

	**Mean**	**SD**	**FOQ**	**OPS**	**CD-RISC**	**SAS**
FOQ	15.561	2.232	1.000			
OPS	3.331	0.542	0.339^**^	1.000		
CD-RISC	63.182	14.921	0.458^**^	0.493^**^	1.000	
SAS	48.970	11.033	−0.170^**^	−0.284^**^	−0.383^**^	1.000

*FOQ, Future Orientation Scale; OPS, Optimization in Primary and Secondary Control Scale; CD-RISC, the Connor–Davidson Resilience Scale; SAS, Zung's Self-Rating Anxiety Scale*.

^***^p < 0.001,

** *p < 0.01*,

^*^p < 0.05.

### The Chain Mediating Analysis

**Figure 2:**
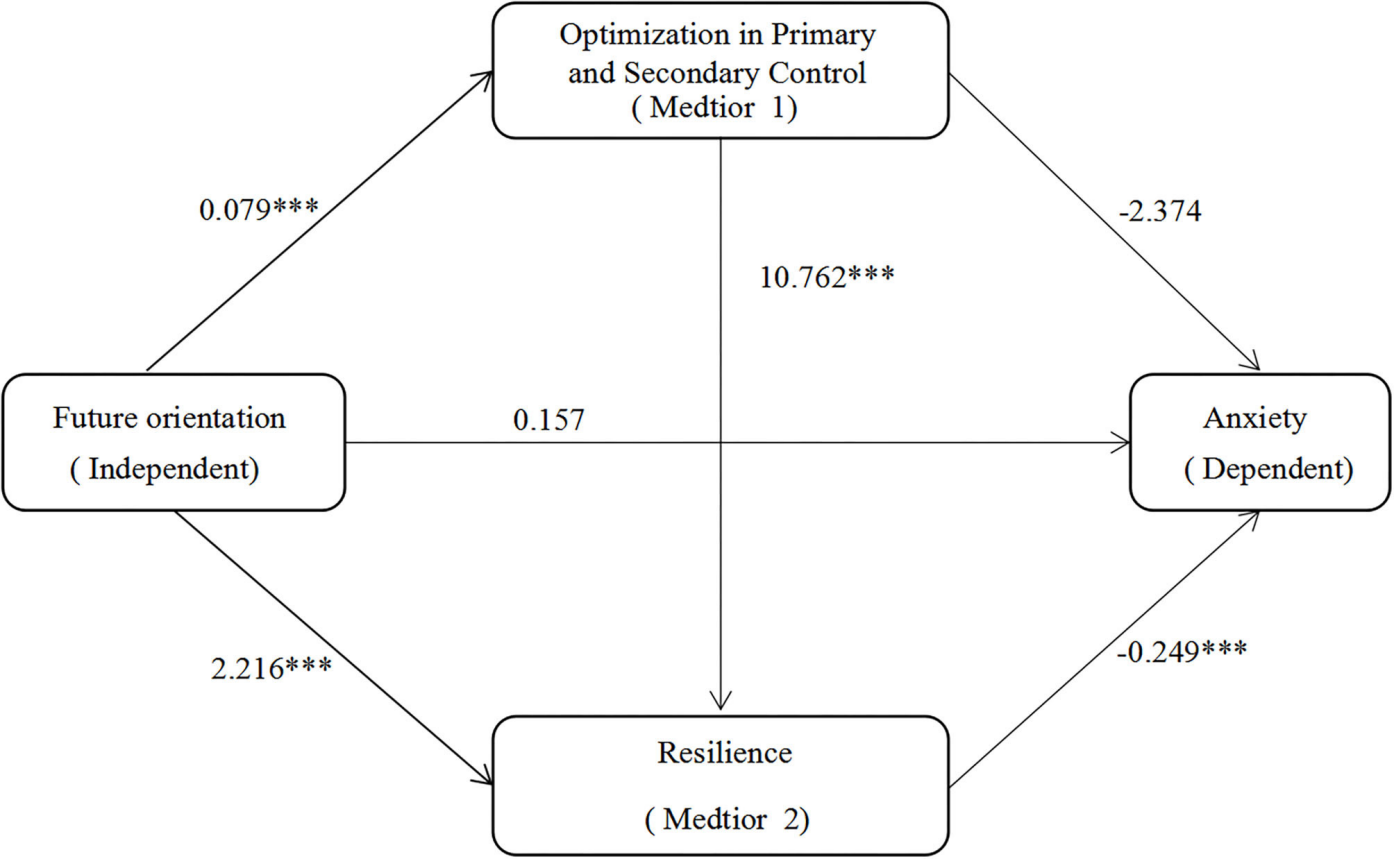
Results of the chain mediation model. Indirect effect 1, Future orientation → Optimization in primary and secondary control → Anxiety; Indirect effect 2, Future orientation → Resilience → Anxiety; Indirect effect 3, Future orientation → Optimization in primary and secondary control → Resilience → Anxiety. ***p < 0.001.

Chain intermediary analyses were performed after controlling for gender and age (Figure 2 and Table 2). Results revealed that the higher future development direction predicts significantly better OPS (B = 0.079, *t* = 5.220, *p* < 0.001). Future orientation (B = 2.216, *t* = 5.609, *p* < 0.001) and OPS (B = 10.762, *t* = 5.976, *p* < 0.001) predicted resilience. Resilience negatively predicted anxiety (B = −0.249, *t* = −5.652, *p* < 0.001). Furthermore, the bootstrap method indicated the significant mediation effects of OPS (Table 3; Effect = −0.188, Boot SE = 0.107, Boot 95% CI = [−0.435, −0.026]), resilience (Effect = −0.552, Boot SE = 0.144, Boot 95% CI = [−0.892, −0.315]), and their chain mediation (Effect = −0.212, Boot SE = 0.067, Boot 95% CI = (−0.373, −0.105]), accounting for 16.934, 49.766, and 19.125% of the total effect, respectively.

**Table 2:** Chain mediation models between future orientation and anxiety (*N* = 528).

**Predictors**	**Model 1 (OPS)**		**Model 2 (CD-RISC)**		**Model 3 (SAS)**	
	**B**	***t***	**B**	***t***	**B**	***t***
Sex	0.105	2.265	−1.363	−1.269	−1.359	−1.469
Age	−0.043	−1.579	0.565	1.015	0.449	1.035
FOQ	0.079	5.220^***^	2.216	5.609^***^	0.157	0.602
OPS			10.762	5.976^***^	−2.374	−1.989
CD-RISC					−0.249	−5.652^***^
*R* ^2^		0.132		0.343		0.165
F		18.414^***^		53.281^***^		15.051^***^

*FOQ, Future Orientation Scale; OPS, Optimization in Primary and Secondary Control Scale; CD-RISC, the Connor–Davidson Resilience Scale; SAS, Zung's Self-Rating Anxiety Scale. The dependent variable in Models 1–3 was separately optimized in primary and secondary control, resilience, and anxiety*.

*** *p < 0.001*;

^**^p < 0.01;

^*^p < 0.05.

**Table 3:** Chain mediating paths between future orientation and anxiety.

	**Effect**	**BootSE**	**BootLLCI**	**BootULCI**	**Relative effect (%)**
Direct effect	0.157	0.26	−0.354	0.667	
Indirect effect 1	−0.188	0.107	−0.435	−0.026	16.93
Indirect effect 2	−0.552	0.144	−0.892	−0.315	49.77
Indirect effect 3	−0.212	0.067	−0.373	−0.105	19.13
Total indirect effect	−0.952	0.168	−1.315	−0.664	85.83

*Indirect effect 1, Future Orientation Scale → Optimization in Primary and Secondary Control Scale → Zung's Self-Rating Anxiety Scale; Indirect effect 2, Future Orientation Scale → the Connor–Davidson Resilience scale → Zung's Self-Rating Anxiety Scale; Indirect effect 3, Future Orientation Scale → Optimization in Primary and Secondary Control Scale → the Connor–Davidson Resilience Scale*.

## Discussion

This study principally investigated the chain mediating path of future orientation and anxiety in the non-infected students during COVID-19 outbreak. The results showed that future orientation lowers anxiety through the indirect paths of OPS, resilience, and the chain mediating path of OPS and resilience.

Previous studies have found that some demographic variables are related to anxiety. For example, female are more anxious than male (53, 54). This can also be because male are more susceptible to stress and therefore are at risk of anxiety and depression, and Young people are more anxious than the old one (56). The deleterious effect of anxiety and depressive symptomatology is tempered by age (52). The children from two-parent families are less anxious than those from single-parent families (57). It may be because two-parent families have better financial security and quality company time. Furthermore, urban residents are more anxious than rural residents (58). This may be due to fiercer competition in the urban economy, which is more likely to cause anxiety.

This result was found to be consistent with the hypothesis that future orientation reduces anxiety through OPS (H1). From the functional perspective of the evolutionary theory, the pursuit of control is an individual's innate biological instinct (8, 59). When the individual's sense of control is threatened and reduced, the uncertainty and disorder make individuals feel anxious. Yet, the OPS is a control strategy for allocating resources to regulate oneself and the environment. The compensatory control theory research suggests that when an individual encounters irreversible factors, the psychological significance of using OPS lies in reducing the psychological discomfort caused by uncertain factors and meeting the needs for structure and order (60). In addition, compared with the individuals with fatalistic and hedonistic time orientation, individuals with future time views produce more positive motives to respond to life changes (61), which reduces future anxiety. Moreover, research from Future Time Perspective (FTP) shows that students with positive future time insight also have a more positive attitude toward their academic tasks (10). The achievement goal theory also believes that in the process of completing academic tasks, students' goals or intentions have a guiding effect on the emotions in the learning situation (62).

Furthermore, this study revealed that future orientation could reduce anxiety by increasing resilience (H2). Block and Kremen (63) reported that positive emotionality is an essential characteristic of resilience, which helps an individual in developing an optimistic attitude toward life and effectively cope with anxiety and adversity (64–66). Moreover, theoretical and empirical studies have indicated that anxiety is related to negative thinking about future (67). Negative cognition affects psychological changes, such as self-regulation (68) and adaptation to life events (67); these psychological changes further exacerbate the anxiety level of students.

This study also revealed that future orientation reduces anxiety of students through the chain mediation of OPS and resilience (H3). Individuals with high future orientation ordinarily have high motivation for achievement (69, 70); people choose adaptive strategies to maintain and continuously stimulate their level of motivation to achieve future goals (67). Additionally, because the choice of strategy makes people more adaptive to life (71), this strategy further leads to less anxiety (72). On the other hand, during the formation and development of resilience, OPS plays a critical intermediary role as a protective factor in reducing the negative impact of unfavorable situations in an individual (29, 30), thereby reducing anxiety.

There are some limitations, for example, we failed to conduct in-depth research and failed to understand other sociological information. Considering that students come from families with guaranteed income may be less anxious, but students come from families with less financial security may be the opposite. In our future research, we aim to use a longitudinal design or experimental paradigm to further support this research hypothesis. Finally, the questions that we aim to explore in our future studies are: how do the protective factors of resilience and anxiety constitute an utterly dynamic system; how does it interact with various risk factors; and how do OPS and resilience stimulate and promote each other, which help students maintain a good state of emotions, abilities, and social interactions in the process of growth?

## Conclusions

Collectively, we explored the protective factors for anxiety. We investigated how students deal with the risk factors after encountering adversity and how their psychological flexibility stimulates and promotes them to achieve a well-adapted developmental state. The findings showed that future orientation reduces anxiety through the indirect paths of OPS and resilience and the chain mediating pathway of OPS and resilience, which provide students the reference suggestions and intervention guidance on reducing anxiety in case of emergencies.

## Data Availability Statement

The raw data supporting the conclusions of this article will be made available by the authors, without undue reservation.

## Ethics Statement

The studies involving human participants were reviewed and approved by The First Affiliated Hospital of Xi'an Jiaotong University. The patients/participants provided their written informed consent to participate in this study.

## Author Contributions

YC: conceptualization, methodology, formal analysis, writing—original draft, and visualization. CL: conceptualization and writing—review and editing. JZ, HX, and CG: conceptualization, project administration, writing—review and editing, and supervision. All authors contributed to the article and approved the submitted version.

## Conflict of Interest

The authors declare that the research was conducted in the absence of any commercial or financial relationships that could be construed as a potential conflict of interest.

## Publisher's Note

All claims expressed in this article are solely those of the authors and do not necessarily represent those of their affiliated organizations, or those of the publisher, the editors and the reviewers. Any product that may be evaluated in this article, or claim that may be made by its manufacturer, is not guaranteed or endorsed by the publisher.
